# Deep View of HCC Gene Expression Signatures and Their Comparison with Other Cancers

**DOI:** 10.3390/cancers14174322

**Published:** 2022-09-03

**Authors:** Yuquan Qian, Timo Itzel, Matthias Ebert, Andreas Teufel

**Affiliations:** 1 Division of Hepatology, Division of Clinical Bioinformatics, Department of Medicine II, Medical Faculty Mannheim, Heidelberg University, 68167 Mannheim, Germany; 2 Department of Medicine II, Medical Faculty Mannheim, Heidelberg University, 68167 Mannheim, Germany; 3 Clinical Cooperation Unit Healthy Metabolism, Center for Preventive Medicine and Digital Health Baden-Württemberg (CPDBW), Medical Faculty Mannheim, Heidelberg University, 68167 Mannheim, Germany

**Keywords:** prognostic, gene expression signatures, hepatocellular carcinoma, specificity, signature generation

## Abstract

**Simple Summary:**

There is a huge gap between the numerous HCC gene signatures and the fact that no one HCC signature has successfully entered clinical practice. The purpose of this study is to explore the specificity of public signatures to HCC as this may be critical for clinical application. For this, we evaluated public HCC signatures using a comparative transcriptomics profiling approach and showed that specificity of current HCC signatures remains challenging, demonstrating the need of standards in gene signature generation and tissue/RNA preparation.

**Abstract:**

Background: Gene expression signatures correlate genetic alterations with specific clinical features, providing the potential for clinical usage. A plethora of HCC-dependent gene signatures have been developed in the last two decades. However, none of them has made its way into clinical practice. Thus, we investigated the specificity of public gene signatures to HCC by establishing a comparative transcriptomic analysis, as this may be essential for clinical applications. Methods: We collected 10 public HCC gene signatures and evaluated them by utilizing four different (commercial and non-commercial) gene expression profile comparison tools: Oncomine Premium, SigCom LINCS, ProfileChaser (modified version), and GENEVA, which can assign similar pre-analyzed profiles of patients with tumors or cancer cell lines to our gene signatures of interests. Among the query results of each tool, different cancer entities were screened. In addition, seven breast and colorectal cancer gene signatures were included in order to further challenge tumor specificity of gene expression signatures. Results: Although the specificity of the evaluated HCC gene signatures varied considerably, none of the gene signatures showed strict specificity to HCC. All gene signatures exhibited potential significant specificity to other cancers, particularly for colorectal and breast cancer. Since signature specificity proved challenging, we furthermore investigated common core genes and overlapping enriched pathways among all gene signatures, which, however, showed no or only very little overlap, respectively. Conclusion: Our study demonstrates that specificity, independent validation, and clinical use of HCC genetic signatures solely relying on gene expression remains challenging. Furthermore, our work made clear that standards in signature generation and statistical methods but potentially also in tissue preparation are urgently needed.

## 1. Introduction

Liver cancer is one of the most frequent, and the fourth most lethal, cancer around the globe [1]. Hepatocellular carcinoma (HCC) is the most common primary hepatic malignant tumor and accounts for more than 80% of all liver cancers worldwide. Despite the advances in therapy, patients with HCC still have poor outcome, especially those at advanced stages [2]. Therefore, early diagnosis and risk stratification of prognosis are of great significance for patients with liver cancer, and initiating effective treatment expeditiously may efficiently improve their prognosis [3]. Unfortunately, real-world utilization of biomarkers to predict clinical outcome of HCC patients by precise classification and specific clinical decision-making remains urgent but still lacking.

In the past two decades, the relevant research communities have made great strides in microarray, high-throughput, and multi-omics technologies. One of the most widely adopted applications of these technologies is to generate a large number of cancer gene expression-associated signatures, including HCC gene signatures [4,5]. Plenty of HCC prognostic gene expression signatures have been described. As one of the pioneering examples, Lee et al. analyzed gene expression profiling data of 91 HCC patients and identified two distinctive subclasses that are highly associated with the survival of patients [6]. Subsequently multiple signatures were reported to be associated with survival [7,8], recurrence [9,10], metastasis [11], and other clinical parameters.

However, even though HCC gene signatures have been studied extensively, none of the published HCC-dependent genetic signatures have entered clinical practice. This is an issue that also remains challenging in other cancer entities, e.g., breast cancer (BC) and colorectal cancer (CRC) [5]. Although many authors addressed the robustness and sensitivity of their molecular-based HCC biomarkers when identifying these gene signatures, they mostly did not widely evaluate the specificity of HCC gene signatures, which is also a crucial aspect of applying HCC gene signatures into clinical routines. In this study, mainly by using four public and commercial gene expression comparison tools, we aimed to explore the specificity of HCC gene signatures, compared it with gene signatures of other cancer entities, and aimed to investigate the difficulties associated with these approaches.

## 2. Material and Methods

### 2.1. Selection of HCC Gene Expression Signatures

**Figure 1:**
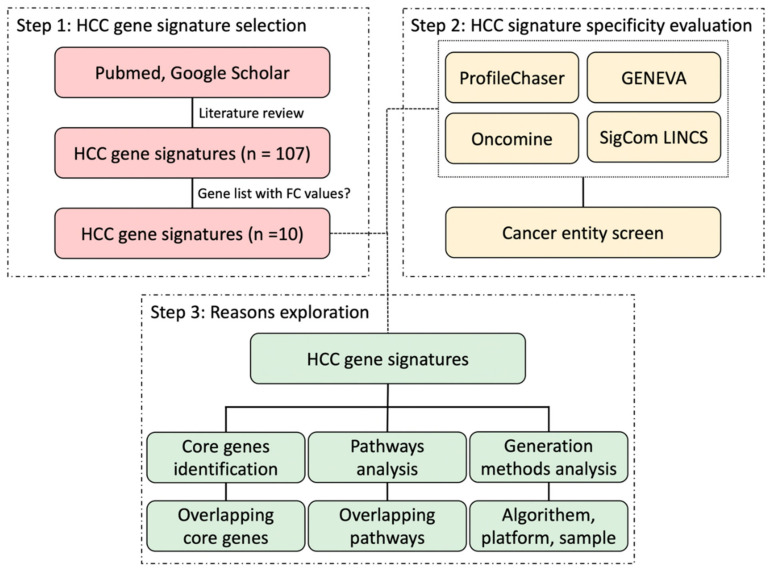
Workflow of this study. FC: fold change.

To select and evaluate the available HCC gene signatures, we first performed literature searches via NCBI PubMed and Google Scholar with the following terms of “gene expression signature” AND “liver cancer” OR “HCC”. Subsequently, we reviewed and evaluated the retrieved articles and considered those gene signatures derived from mRNA expression profiling studies. In this manuscript, we used primarily gene signatures for which fold-change values of differential expression analysis were available in the published articles according to the requirements of ProfileChaser. As a result, 10 HCC gene signatures were selected for our follow-up study and they are all related to prognosis. The workflow of this study is presented in Figure 1.

### 2.2. ProfileChaser Analysis

The publicly available webtool ProfileChaser allows for mining pre-analyzed Gene Expression Omnibus (GEO) profiles for similar differentially regulated transcriptional programs [12]. As the default setting for querying is to use an existing GEO Dataset or upload a dataset, we modified ProfileChaser in order to be able to upload gene sets/signatures directly and have ProfileChaser assign similar pre-analyzed profiles to our signatures of interests. Those results with a q-value < 0.05 were screened for different cancer categories.

### 2.3. Oncomine Concept Association Analysis

In addition, we used the commercialized platform Oncomine, which provides several standardized bioinformatics analysis functions including differential expression analysis, co-expression analysis, and outlier analysis [13]. Gene signatures can be handled as concepts, which are defined as sets of genes representing some biological characteristics in the Oncomine Research Premium Edition. We uploaded our selected HCC, BC, and CRC gene signatures to Oncomine as different concepts, and performed association analysis by comparing them to all other pre-extracted concepts in Oncomine. The threshold odds ratio and *p*-value were set to 3 and 0.0001, respectively, defining even more stringent searches compared to standard parameters. The gene expression comparison was derived from cancer tissue and normal tissue. Like in ProfileChaser, different cancer categories and the number of matched datasets were counted from the results.

### 2.4. GENEVA Gene Signature Query

GENEVA (https://genevatool.org/ (accessed on 24 June 2021)) is short for Gene Expression Variance Analysis, which enables users to query relevant RNA-seq datasets of a gene or a gene signature by identifying the variance of gene expression [14]. The GENEVA score compares the variance of a gene in combination of the regression coefficient based on the experimental setting. To take the global variance of the data set into account, the score is divided by the average variance of all genes. Therefore, we focused on the datasets matched with a GENEVA score > 1 for each HCC gene signature. Same as before, we screen different cancer entities in the results.

### 2.5. Sigcom LINCS Gene Signature Search

The signature-searchable platform Sigcom LINCS (https://maayanlab.cloud/sigcom-lincs/#/SignatureSearch/UpDown (accessed on 18 June 2021)) contains 1,536,533 gene expression signatures integrated from the Library of Integrated Network-Based Cellular Signatures (LINCS), the Genotype-Tissue Expression (GTEx), and GEO [15]. Up-down enrichment mode was adopted for signature enrichment analysis, and then different cancer entities were screened in the automatic human GEO RNA-seq signature mimickers, namely matching signatures with a positive z-score (enrichment score).

### 2.6. Core Genes and Pathways Identification

To explore whether HCC gene signatures have common core genes, we first constructed a protein-protein interaction (PPI) network of each HCC gene signature using the STRING database, and then identified hub genes via the plug-in cytohubba of Cytoscape software (version 3.8.2, Cytoscape Team). Finally, we checked if there are any overlapping genes between those core genes of each HCC gene signature.

Kyoto Encyclopaedia of Gene and Genomes (KEGG) and Reactome pathway enrichment analyses were performed together by using WebGestalt (http://www.webgestalt.org (accessed on 1 August 2021)). We adopted Over-Representation Analysis as the method of interest, and selected protein-coding genome as the reference set when running Webgestalt. For each gene signature, we selected significant pathways with a false discovery rate (FDR) <0.05 and the top 10 most significant pathways based on enrichment ratios, respectively. Then, like core gene results, we compared the pathway results of gene signatures with each other to figure out the common pathways.

### 2.7. Signature Generation Methods

Considering that HCC gene signatures were identified from different study groups using varying platforms and applying different screening algorithms, we compared three important and distinct aspects to analyze the methods of HCC gene signature derivation: gene expression platform, algorithm to screen signature genes, and source of the samples.

## 3. Results

### 3.1. Selected HCC Signatures Based on ProfileChaser

After screening HCC gene signatures according to the requirement of applying ProfileChaser, 10 HCC gene signatures including 2008 Coulouarn [16], 2009 Kaposi-Novak [17], 2010 Roessler [10], 2010 Woo [18], 2010 Andersen [19], 2012 Roessler [20], 2016 Villa [21], 2017 Chen [11], 2019 Guan [22], and 2020 Yi [23] were finally included in the study (Table 1). These gene signatures were published between 2008 and 2020, and the number of genes ranged from 5 to 625. The types of signatures are all prognostic gene signatures, including survival, metastasis, recurrence, growth, and malignant transformation. The differential analyses for the generation of these signatures are diverse, such as a comparison between good survival group and poor survival group, HCC tumor tissue versus non-tumor tissue, early-stage versus late-stage, and metastasis versus metastasis-free.

### 3.2. ProfileChaser and Oncomine Query Results of HCC Signatures

As for the query results of HCC gene signatures run in ProfileChaser, a web server was used that allows for content-based gene expression search with a user-supplied experiment. Half of these 10 gene signatures matched liver cancer profiles. In addition to liver cancer, these five HCC signatures also matched other cancers, such as lung cancer, breast cancer, and renal cancer. The remaining five signatures, 2009 Kaposi-Novak, 2010 Roessler, 2012 Roessler, 2016 Villa, and 2020 Yi, did not match any cancer profiles using ProfileChaser, no matter liver cancer or other types of cancer. Among the matched cancer types, liver cancer, renal cancer, colorectal cancer, and breast cancer were the most frequent (Table 2).

In Oncomine, HCC gene signatures matched more diverse cancer types and more data sets. At least 18 cancer types including liver cancer were matched by these 10 HCC gene signatures. Specifically, the 2009 Kaposi-Novak, 2010 Roessler, 2016 Villa, and 2020 Yi signatures only matched only one cancer type or nothing. The other 6 HCC gene signatures all matched 3–12 cancer types including liver cancer, colorectal cancer, renal cancer, breast cancer, and sarcoma. Besides, we noted that not only cancer types but also more datasets were fetched in Oncomine than in ProfileChaser. For example, 2019 Guan matched 5 liver cancer profiles, 8 lung cancer profiles, 3 colorectal cancer profiles, 10 breast cancer profiles, and 4 sarcomas profiles (Table 2).

### 3.3. GENEVA and Sigcom LINCS Query Results of HCC Signatures

The query results of yet two additional, publically available profiling tools GENEVA and Sigcom LINCS are presented in Table 3. Overall, the vast majority of HCC gene signatures were found to exhibit rather universal significance across various cancer types in either GENEVA or SigCom LINCS. Among them, six HCC gene signatures namely 2008 Coulouarn, 2010 Woo, 2010 Andersen, 2012 Roessler, 2019 Guan, and 2020 Yi retrieved liver cancer and other cancers in both GENEVA and Sigcom LINCS. In addition, signatures 2009 Kaposi-Novak and 2010 Roessler only retrieved definite matches for human cancer entities in SigCom LINCS, while 2016 Villa only obtained matches from GENEVA. Signature 2017 Chen did not see any matching cancer types in both GENEVA and SigCom LINCS.

### 3.4. Similarities and Differences of Query Results between the Four Bio-Tools

**Figure 2:**
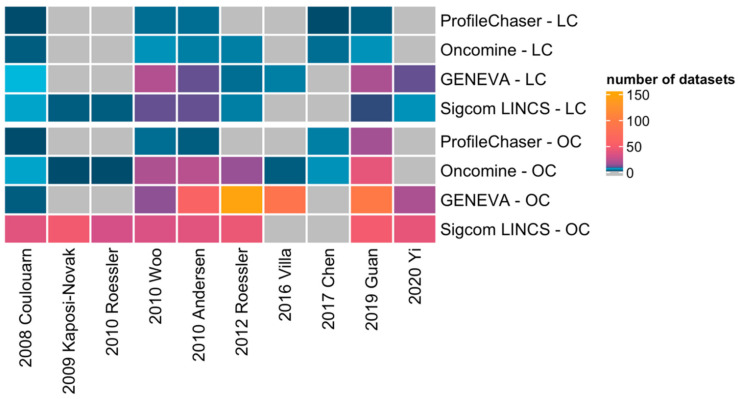
HCC gene signature search results of four bio-tools. LC: Liver Cancer, OC: Other Cancers. The color represents the number of datasets matched in each tool; grey mean no datasets matched [10,11,16,17,18,19,20,21,22,23].

After integrating the results from the four profiling tools (Figure 2), we demonstrated that all of the HCC gene signatures matched different cancer profiles in at least two comparison tools, indicating that these HCC gene signatures are not specific enough for HCC, especially the signatures distinguishing between good survival and poor survival samples. From Table 2 and Table 3, we observed that the most common types of cancers matched are similar, including liver cancer, colorectal cancer, and breast cancer. We also found that the tools that match liver cancer can basically match other cancers, and the similarity search results for ProfileChaser and Oncomine are relatively similar, as are those for GENEVA and Sigcom LINCS. Furthermore, we also found that four of five HCC gene signatures that did not have any match in ProfileChaser, 2009 Kaposi-Novak, 2010 Roessler, 2016 Villa, and 2020 Yi, also matched only one type of cancer or did not match any cancer entity in Oncomine. In addition, HCC signatures in GENEVA and SigCom LINCS almost always match a wide range of tumor types, if they can retrieve a match at all.

Certainly, the striking differences in the matching results of the four tools must be highlighted. Not only the proportion of the 10 HCC gene signatures that successfully screened for tumor expression profile matches varied across the four tools, but the tumor types and datasets matches also differed drastically. In comparison with ProfileChaser, Oncomine presented better mining of HCC gene signatures in terms of a matching rate of 90% (9/10) vs. 50% (5/10), as well as matching to more diverse cancer types and a wider range of expression profiles. Moreover, at least five HCC gene signatures showed large differences between GENEVA and SigCom LINCS runs in terms of whether they could match to tumor entities and the numbers of matched tumor types.

### 3.5. Oncomine Results of Breast Cancer and Colorectal Cancer Gene Signatures

In order to compare their performance and challenge tissue/entity specificity, we selected available BC and CRC gene signatures and evaluated them in comparison to HCC gene signatures. We included five BC gene signatures: Oncotype DX Breast [24], MammaPrint [25], Endopredict [26], Prosigna/PAM50 [27], and Breast Cancer Index [28,29], and two CRC gene signatures: Oncotype DX Colon [30] and ColoPrint [31].

Five of these seven gene signatures retrieved associated cancer types in Oncomine (Table 4). Among the five gene signatures, except for Oncotype DX Colon that matched 4 types of cancer, other gene signatures matched 12, 13, 16, and 19 types of cancers respectively. Endopredict and ColoPrint did not match any profiles in Oncomine. BC, gastric cancer, sarcoma, CRC, bladder cancer, leukemia, etc. are the most frequent matched tumor types.

### 3.6. Common Core Genes and Common Pathways between HCC Gene Signatures

We confirmed the core genes of each gene signature by using PPI and Cytoscape and checked if there are any overlapping genes between these HCC gene signatures. For most signatures, 3–13 genes were identified as core genes. In contrast, for the 2020 Yi signature, no core genes could be identified. However, the core genes of these signatures did not have any overlap (Table 5).

**Figure 3:**
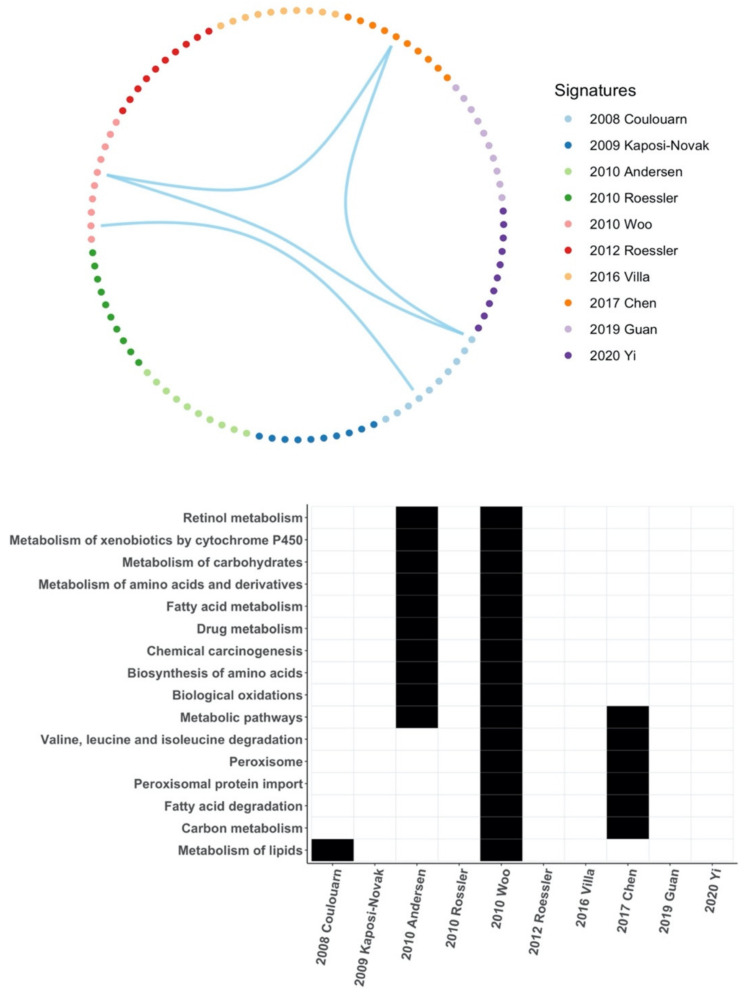
Common pathways of HCC gene signatures. **Above**: Of the top 10 pathways in each HCC gene signature based on the enrichment ratios, only two pathways overlap between three HCC gene signatures. **Down**: There are 16 overlapping pathways among the significant pathways screened by each HCC gene signature based on FDR < 0.05, but almost all of them overlapped between only two HCC gene signatures [10,11,16,17,18,19,20,21,22,23].

Meanwhile, based on the enrichment ratios, we selected the top 10 significant pathways for follow-up analysis. Similarly, we just found two significant overlapping pathways between three HCC gene signatures, namely the metabolism of lipids between 2008 Coulouarn and 2010 Woo, and the metabolic pathways between 2008 Coulouarn, 2010 Woo, and 2017 Chen. In addition, we also used FDR < 0.05 as the cutoff criterion for significant pathway screening. After comparing the significant pathways enriched in each HCC gene signature, we found 16 pathways that were enriched in at least two HCC gene signatures. However, 15 of these 16 pathways were enriched in only two gene signatures, mainly between 2010 Woo and 2010 Andersen or 2010 Woo and 2017 Chen (Figure 3).

### 3.7. Signature Generation Differences

After observing the above phenomenon, we tried to figure out whether there are differences in the methods of producing these HCC gene signatures. From the platform of gene expression profiling, the algorithm involved in the signature gene selection, and the sample source of these HCC gene signatures, we found that the methods of generating HCC gene signatures varied massively (Table 6).

As for the algorithms for signature generation, there are mainly five methods involved: Differential gene expression, Cox regression, External signature, Unsupervised hierarchical clustering, and gene co-expression network analysis. The platforms are mainly involving Affymetrix, Illumina, Agilent, and Custom NCI array, while the origin of samples involved in the generation of these gene signatures differs from Asia, Europe, and North America. If we combine these three factors, each gene signature is identical to other signatures. Even if they are the same in the source of samples, i.e., 2008 Coulouarn, 2010 Roessler, 2010 Andersen, and 2017 Chen, they use different platforms and applied four different approaches.

## 4. Discussion

The profound heterogeneity of HCC is becoming increasingly clear. The intra-tumor heterogeneity of HCC leads to the lack of robustness and reproducibility of molecular biomarkers [32], and meanwhile random gene sets show prognostic power for patients with HCC [33]. This may also be one of the major obstacles in establishing valid gene expression signatures for the diagnosis, prognosis, and response to treatment in patients with HCC.

Gene expression profiling creates a panoramic view of cellular function by measuring the expression of thousands of genes at once, which makes it feasible to detect and classify genetic changes in cells in the form of gene expression signatures [5]. Although the advancements have been made, successful translation of gene expression profiling into clinical applications remains challenging. The limitations include not only the need for further optimization of tissue preparation and storage procedures, but also the need for sufficient bioinformatics strategies and standardized independent validation of gene expression signatures [4].

However, up to now no global comparison of the available gene expression signatures has been attempted. Therefore, by means of implementing four comparison bio-tools, we successfully validated in this study that current HCC gene signatures are unspecific for HCC, which may have several underlying reasons.

In this study, the results of similar expression profiles search vary among the four profiling tools. On one hand, how the four tools perform gene signature query is quite different: ProfileChaser compares gene expression profiles by weighted correlation coefficient, Oncomine performs association analysis, GENEVA identifies the variance of gene expression, and Sigcom LINCS does signature enrichment analysis. On the other hand, the composition of databases on which the four tools are based is also different: while datasets in ProfileChaser and Oncomine are composed of microarray data, GENEVA and Sigcom LINCS perform signature search based on RNA-seq data.

Unlike HCC gene signatures, some BC and CRC gene signatures are already commercially or clinically available. Our study shows that not only HCC gene signatures, but the majority of these available BC and CRC gene signatures (5/7) also got many different cancer matches after running in Oncomine. This suggests that even though these gene signatures have been commercialized or entered the clinic, they are still not specific enough. In fact, currently available gene signatures for early and intermediate stages of CRC should only be used in specific clinical settings due to the lack of a plausible biological interpretability and have no predictive value of treatment benefit [5,34]. Although the promising CRC classifier based on gene expression, the consensus molecular subtypes (CMS) classification system, shows prognostic value in intermediate and advanced-stage CRC, there is still a lack of standardization and a requirement for bioinformatics resources [5]. BC has a leading edge in the clinical application of gene signatures, but there is still debate about their use in BC, especially in early BC with positive lymph nodes [5]. In addition, Manjang and co-workers also demonstrated that the prognostic BC gene signatures lack a clear biological meaning [35], suggesting us to notice the divergences at the level of gene patterns and gene expression profiles.

In order to overcome those obstacles, recent studies revealed that long noncoding RNAs (lncRNAs), due to abnormalities in chromatin modification and alternative splicing, play an important role in tumor development and progression and are more likely cancer-type specific compared with protein-coding genes [36,37,38]. The gene signatures included in our study were all protein-coding genes, as these were commonly utilized in gene expression profiling in the past and available in respective databases and evaluation tools. However, further exploration of lncRNA signatures may provide additional insights into molecular mechanism, ultimately leading to progress in the management of HCC.

Core genes or hub genes are at the core of the regulatory network and play an important role in the biological classification of samples by gene signatures. In this study, all core genes were unique to each HCC gene signature and only a few signal pathways overlapped between few HCC gene signatures, which indicates that they may produce very different biological classifications. This accounts for the differences between those HCC gene signatures.

Benefiting from the bioinformatic technology advances, the scientific community has developed a vast number of methods to generate gene signatures. Different platforms, algorithms, and sources of samples play an important role in the generation of cancer gene signatures. In our study, taking the above three factors into consideration, all the selected HCC gene signatures have different generation methods. Meanwhile, these technical differences and variance in samples have led to biological limitations of HCC gene signatures. A standardized method and procedure are needed to construct more interpretable and comparable gene signatures.

Our study demonstrates that current HCC genetic signatures are not good enough for prognosis and independent validation of HCC gene signatures remains challenging, which may be critical for HCC gene signatures to enter clinical application. Overall, due to the heterogeneity of HCC, our data suggest the necessity of standards, not only in data format and tissue storage, but also in signature generation, statistical methods, and independent validation.

Due to enabling reproducible re-analysis of functional genomics data, the scientific community has long been aware of the need to standardize the data format when describing a microarray or sequencing study. The Functional Genomics Data (FGED) Society proposed two recording and reporting standards successively: the Minimum Information About a Microarray Experiment (MIAME) [39] and the Minimum Information about a high-throughput SEQuencing Experiment (MINSEQE) [40]. The two guidelines both emphasized the importance of providing the following information to make the data understandable and reusable: (1) raw data and final processed data; (2) general information about the experiment; (3) sample annotation, the experimental factors, and their values; (4) laboratory and data processing protocols; and (5) sample data relationships [40,41]. Data repositories such as GEO and ArrayExpress both employed MIAME and MINSEQE as standards for data depositing, facilitating data submission/sharing and usage.

Sample storage is another critical factor for gene expression profiling as extraction of high-quality RNA from the samples is a prerequisite for reliable measurement results [42,43]. While cryopreserved cancer tissue had no adverse effect on RNA quality, RNA degradation depended on the time the samples were not frozen [44]. Uniform standards for sample storage are not well established, which may also account for the inconsistent findings among different studies, but a few basic guidelines are recommended. First, patient samples need to be frozen and stored at −80 °C as soon as possible, preferably with cold ischemia less than 1 h [44]. Second, RNA stabilization reagents, i.e., RNAlater, are recommended to preserve RNA integrity during frozen storage [45]. Third, samples should be transported on ice or in liquid nitrogen to avoid significant degradation of RNA quality before freezing them. Fourth, samples can be frozen in many small sections and stored in separate partitions to reduce the effects of repeated freezing and thawing and temperature fluctuations.

A variety of gene signature generation methods exist in different studies and they are evolving more and more sophisticated, contributing to the limited reproducibility and comparability of HCC gene signatures. No standards for gene signature generation algorithm have been established, yet we would like to propose that the algorithms should meet two criteria: (1) the algorithms need to select genes that are not only statistically significant but also biologically meaningful, and (2) the algorithms should maintain their effectiveness on different sequencing platforms and samples from different sources to rule out that they are platform-specific or sample-specific. In addition, the importance of independent validation is always in need of emphasizing.

## 5. Conclusions

So far, HCC gene signatures are not so specific and show algorithmic dependency in validation. Even though some gene signatures for BC and CRC are commercially or clinically available, most of them also lack specificity. There are a few common pathways between these gene signatures and no common core genes, and the different generation methods of these HCC gene signatures may be the main reasons for this phenomenon. We therefore strongly advocate for implementing standards in gene expression signature generation and tissue/RNA preparation.

## Figures and Tables

**Table 1:** Ten selected HCC gene signatures for validation.

Signature	Clinical Outcome	No. Genes	Comparison
2008, Coulouarn [16]	Survival	249	early vs. late
2009, Kaposi-Novak [17]	Malignant Transformation	85	Dysplastic nodules vs. cirrhotic (regenerative) nodules
2010, Roessler [10]	Metastasis, Recurrence	161	metastasis vs. metastasis-free
2010, Woo [18]	Survival	625	cholangiocarcinoma-like HCC vs. other HCCs
2010, Andersen [19]	Survival	110	poor prognosis vs. better prognosis
2012, Roessler [20]	Survival	10	good survival vs. poor survival
2016, Villa [21]	Growth, Survival	5	fast vs. slow growing tumors
2017, Chen [11]	Metastasis	6	HCC tumor tissue vs. non-tumor tissues
2019, Guan [22]	Survival	55	good prognostic group vs. poor prognostic group
2020, Yi [23]	Survival	14	with vs. without vascular invasion

**Table 2:** ProfileChaser and Oncomine results of HCC gene signatures.

	2008, Coulouarn [16]		2009, Kaposi-Novak [17]		2010, Roessler [10]		2010, Woo [18]		2010, Andersen [19]		2012, Roessler [20]		2016, Villa [21]		2017, Chen [11]		2019, Guan [22]		2020, Yi [23]	
	On	Pro	On	Pro	On	Pro	On	Pro	On	Pro	On	Pro	On	Pro	On	Pro	On	Pro	On	Pro
Liver	2	1					5	3	4	3	4				3	1	5	2		
Lung											1						8	2		
Colorectal							1	1	4	1	3						3	1		
Breast		1	1				4		1		2						10	7		
Kidney	1						3	1	4	1			2		4	4		1		
Lymphoma	2								3									1		
Leukemia	1										1							1		
Sarcoma	1						2	1	4								4	1		
Glioma																		1		
Esophageal							2		1								3			
Cervical							1										1			
Gastric							2		1								3			
Head and Neck							1		1		1									
Ovarian									1		1						1			
Melanoma											1							1		
Prostate							1		1		2									
Pancreatic															1		1			
Bladder					1						1						3			
Other Cancer	1						2		1		1						2			

On: Oncomine, Pro: ProfileChaser.

**Table 3:** GENEVA and Sigcom LINCS results of HCC gene signatures.

	2008, Coulouarn [16]		2009, Kaposi-Novak [17]		2010, Roessler [10]		2010, Woo [18]		2010, Andersen [19]		2012, Roessler [20]		2016, Villa [21]		2017, Chen [11]		2019, Guan [22]		2020, Yi [23]	
	GE	Sig	GE	Sig	GE	Sig	GE	Sig	GE	Sig	GE	Sig	GE	Sig	GE	Sig	GE	Sig	GE	Sig
Liver	7	6		2		2	20	9	9	9	3	4	4				18	8	9	5
Lung	2	1				1	2	3	7	3	7	2	3				6	2	3	2
Colorectal		5		2		1		3	4	3	6	3	3				5	3	2	4
Breast		4		7		7	1	5	8	9	17	11	10				18	8	5	4
Kidney		2					1	3	1	2	5		4				1	3	1	2
Lymphoma				1		1				4	6	1	1					1		1
Leukemia		2		5		1			8	1	21	3	6				9	4	2	2
Sarcoma		1		1		2		2	4		16	3	4				5	1		2
Glioma		1		1		2			2		7		4				4	2		3
Esophageal		1						1		2		1						1		1
Cervical		1		2		2			1		1		1					1		3
Gastric		2		1			1	2	1	2			1				3	1		1
Head and Neck		3		4		5		4	1	2	10	3	3					5		4
Ovarian				1				1	1	1	1	1	1				2		1	1
Melanoma		1		2		3			2	1	5	3	8				7			1
Prostate		2		5		1	3		6	1	7	2	7				19			1
Pancreatic		3		1		1		1	4		7		3				1	1		
Bladder													2					2		1
Other Cancer		8		13		3	5	8	9	6	30	9	27				15	12	4	7

GE: GENEVA, Sig: Sigcom LINCS.

**Table 4:** Oncomine results of breast cancer and colorectal cancer.

	Oncotype DX Breast	MammaPrint	Endopredict	Prosigna/PAM50	Breast Cancer Index	Oncotype DX Colon	ColoPrint
Bladder	2	2		3	3		
Brain and CNS		4		4			
Breast	6	12		20	6	4	
Cervical	2	3		3	1		
Colorectal		9		15	6	4	
Esophageal	2	3		2	1		
Gastric	2	5		6	3	1	
Head and Neck	4	8		12			
Kidney		2					
Leukemia	1	2		3	2		
Liver		3		4	3		
Lung	7	8		17	9		
Lymphoma	2			4		1	
Melanoma				2			
Other cancer	1	4		7	3		
Ovarian	2	3		6	1		
Pancreatic	1	2		2			
Prostate				2			
Sarcoma	3	9		11	6		

**Table 5:** Common core genes of HCC gene signatures.

2008, Coulouarn [16]	2009, Kaposi-Novak [17]	2010, Roessler [10]	2010, Woo [18]	2010, Andersen [19]	2012, Roessler [20]	2016, Villa [21]	2017, Chen [11]	2019, Guan [22]	2020, Yi [23]
SQLE	NUBPL	RAD50	FGA	RPS3A	SH2D4A	ESM1	AGXT	STIL	
HMGCS1	CIAO1	FEN1	C8A	RPS18	CCDC25	DLL4	DAO	RAD51AP1	
SREBF2	ISCA2	RPA2	CPB2	RPL27A	SORBS3	ANGPT2	EHHADH	CDC20	
MSMO1		RFC5	F11	RPL3	PROSC		ABAT	CEP55	
CYP51A1		GTF2H1	SERPINA10	RPS25			ALDH6A1	POLE2	
IDI1		CHEK1	FETUB	RPS12				SPC24	
FDPS		GTF2H4	HRG	RPS17				CCNB1	
DHCR24			F13B	RPS14				KIF20A	
LDLR			FTCD	RPL13A				CDCA3	
DHCR7			SPP2	RPL9				CDT1	
				RPL35A					
				CCT2					
				RPL10A					

**Table 6:** Algorithms involved in the generation of HCC gene signatures.

Signature	Algorithm for Signature Generation	Platform	Origin of Samples
2008, Coulouarn [16]	Differential gene expression	Custom NCI array	America, Asia, Europe
2009, Kaposi-Novak [17]	Differential gene expression	Custom NCI array	Europe
2010, Roessler [10]	Cox regression	Affymetrix HG-U133A, Custom NCI array	America, Asia, Europe
2010, Woo [18]	Differential gene expression	Affymetrix HG-U133A	Asia
2010, Andersen [19]	External signature	Illumina humanRef-8	America, Asia, Europe
2012, Roessler [20]	Unsupervised hierarchical clustering	Affymetrix HG-U133A, Agilent-014698 Human Genome CGH Microarray 105A	America, Asia
.2015, Villa [21]	Cox regression	Agilent-014850 Whole Human Genome Microarray 4 × 44K G4112F	Europe
2017, Chen [10]	gene co-expression network analysis	Affymetrix HG-U133A, Affymetrix HG-U133_Plus_2, IlluminiaHiseq	America, Asia, Europe
2019, Guan [22]	Cox regression	Illumina HumanHT-12 V4.0	Asia
2020, Yi [23]	Cox regression	Illumina Hiseq	NA

NA: not available, NCI: National Cancer Institute.

## Data Availability

Data supporting the reported results can be obtained from the corresponding author.
